# Multiyear Surveillance for Avian Influenza Virus in Waterfowl from Wintering Grounds, Texas Coast, USA

**DOI:** 10.3201/eid1608.091864

**Published:** 2010-08

**Authors:** Pamela J. Ferro, Christine M. Budke, Markus J. Peterson, Dayna Cox, Emily Roltsch, Todd Merendino, Matt Nelson, Blanca Lupiani

**Affiliations:** Texas A&M University, College Station, Texas, USA (P.J. Ferro, C.M. Budke, M.J. Peterson, D. Cox, E. Roltsch, B. Lupiani); Texas Parks and Wildlife Department, Bay City, Texas, USA (T. Merendino, M. Nelson); 1 Current affiliation: Ducks Unlimited, Texas Gulf Coast, Richmond, Texas, USA.

**Keywords:** Avian influenza, wild ducks, migratory waterfowl, surveillance, Texas, waterfowl, wintering grounds, zoonoses, research

## Abstract

We studied the prevalence of influenza A virus in wintering waterfowl from the Central Flyway on the Gulf Coast of Texas. Of 5,363 hunter-harvested migratory and resident waterfowl and wetland-associated game birds sampled during 3 consecutive hunting seasons (September–January 2006–07, 2007–08, and 2008–09), real-time reverse transcription–PCR detected influenza A matrix sequences in 8.5% of samples, H5 in 0.7%, and H7 in 0.6%. Virus isolation yielded 134 influenza A viruses, including N1–N9, H1–H7, H10, and H11 subtypes. Low-pathogenicity H7 subtype was isolated during January, September, and November 2007 and January 2008; low-pathogenicity H5 subtype was isolated during November and December 2007.

## Abstract 2

This surveillance can help in assessments of the prevalence of wild animal-to-human transmission.

Wild waterfowl, primarily species in the orders Charadriiformes and Anseriformes (*1*), are natural reservoirs for type A influenza viruses. These viruses, which are occasionally transmitted to other species, including humans, poultry, and swine, result in subclinical to highly pathogenic diseases. Two subtypes (H5 and H7) have been most frequently associated with high pathogenicity in poultry and are of considerable interest to the poultry industry and to researchers who study avian influenza viruses (AIVs) (*2**–**4*). The migratory nature of many waterfowl species and the persistence of AIV in them present a potential vehicle for global dissemination of influenza viruses, as well as a constant source of viruses and genetic material for new pandemic strains. Preventing the introduction and adaptation of wild bird–origin AIVs to other susceptible species is an efficient strategy for minimizing the effects of AIV on global health and the global economy (*5**,**6*). Thus, surveillance in reservoir species is crucial for identifying viruses and gene pools with interspecies and intraspecies transmission potential.

In North America, migratory birds use 4 major flyways: Pacific, Central, Mississippi, and Atlantic (www.flyways.us). Three flyways (Pacific, Mississippi, and Atlantic) are well represented in the literature that addresses AIV surveillance (summarized in [*7*]); however, data are limited for the Central Flyway (*8**–**10*). Approximately 90% of waterfowl that use the Central Flyway winter in Texas. Of these, ≈10 million ducks and geese winter in wetlands throughout the state, whereas 1–3 million ducks and >1 million geese winter along the Texas Gulf Coast (*11*). Before the implementation of surveillance programs to detect subtype H5N1 highly pathogenic AIV, few surveillance studies included migratory waterfowl on their wintering grounds or nonmigratory waterfowl during winter, particularly for the Texas–Louisiana Gulf Coast, where most studies were limited to just a few waterfowl species and limited by time of year and number of years studied (*8**,**9**,**12**,**13*). Although the US Interagency Strategic Plan for the Early Detection of Highly Pathogenic Avian Influenza H5N1 has extensively sampled waterfowl across all flyways, the program focuses on detection of subtype H5N1 virus; thus, only information pertaining to this subtype is publicly available (*14*). To understand the ecology, natural history, and evolution of influenza viruses, long-term surveillance studies are needed, particularly those that investigate waterfowl in understudied areas, such as wintering grounds. Long-term surveillance is even more important in areas where commercial poultry operations and migratory waterfowl stopover or wintering areas overlap (*15*).

We recently reported AIV prevalence, as determined by real-time reverse transcription–PCR (rRT-PCR) and virus isolation, from a multiyear surveillance project (September 2005–January 2009) of hunter-harvested waterfowl in the Texas mid–Gulf Coast region (*16*). We found little variation in overall AIV prevalence within or between seasons, except for 1 season (2007–08) when the overall prevalence was higher (*16*). The objectives of the current study were to 1) determine subtype diversity of AIV in both migratory and resident waterfowl populations (mostly ducks and geese) to which humans may be exposed and 2) compare prevalence and subtype diversity of AIV among species, according to age and sex, focusing on the Texas mid–Gulf Coast region during early fall and winter, which coincides with the regional hunting season.

## Methods

### Sample Collection and Analysis

During 2006–09, cloacal swab samples were collected from hunter-harvested waterfowl (*17*) and other wetland-associated game birds (*18*) during 3 consecutive hunting seasons: September 2006–January 2007 (season 1), September 2007–January 2008 (season 2), and September 2008–January 2009 (season 3) at 4 state wildlife management areas (WMAs) along the Gulf Coast of Texas: Justin Hurst WMA in Brazoria County, Mad Island WMA in Matagorda County, Guadalupe Delta WMA in Calhoun County, and Matagorda Island WMA in Calhoun County (Figure). Trained field personnel identified the species, sex, and age (when possible) of the bird on the basis of plumage (*19*). The bird’s age was recorded as adult, if it was not the bird’s hatch-year, and juvenile, if it was the bird’s hatch-year. Waterfowl species and areas sampled reflected hunters’ choices and personnel available to collect swabs on sampling days. Data from all 4 WMAs were combined for analysis.

**Figure:**
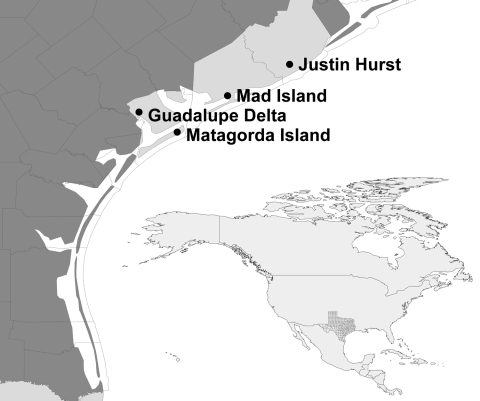
Locations of state wildlife management areas where samples were collected from waterfowl for avian influenza virus surveillance, Texas mid–Gulf Coast, USA, September–January 2006–07, 2007–08, and 2008–09. Inset shows location of Texas (shaded).

All samples were collected, processed, and tested as previously described (*8**,**16*). Briefly, all samples (N = 5,363) were screened for AIV by AIV-matrix rRT-PCR, and virus isolation was performed on all 455 rRT-PCR–positive samples and 3,664 rRT-PCR–negative samples. All rRT-PCR–positive samples were screened for H5 and H7 subtypes by rRT-PCR by using the AgPath-ID One-Step RT-PCR Kit (Ambion, Inc., Austin, TX, USA) and an ABI 7500Fast Real-time PCR System (Applied Biosystems, Inc., Foster City, CA, USA) in a 25-µL final reaction volume. Primers and probes for the M and H5 (*20**,**21*) and H7 subtypes (*21**,**22*) were those previously described. All AIV isolates were submitted to the National Veterinary Services Laboratory (NVSL; Ames, IA, USA) for subtyping by hemagglutination (HA) and neuraminidase (NA) inhibition tests and screening for the presence of the N1 gene by rRT-PCR. Additionally, all H5 and H7 isolates were pathotyped at NVSL by analysis of the amino acid sequence at the HA protein cleavage site.

### Statistical Analysis

We previously documented that prevalence estimates calculated on virus isolation following a positive AIV-matrix rRT-PCR provided results nearly identical to those obtained by performing both tests in parallel (*16*); for this reason, we calculated apparent prevalence by dividing the number of virus isolation–positive samples (after a positive rRT-PCR result) by the total number of samples collected and tested by rRT-PCR (*16*).

Pearson χ^2^ analyses were used to evaluate differences in AIV-infected proportion by sex (drake vs. hen), age (adult vs. juvenile), species of waterfowl, and hunting season of collection (seasons 1, 2, 3). Fisher exact test was used instead of χ^2^ when >1 cells were expected to have a frequency of <5. A p value <0.05 was considered significant. Wald 95% confidence intervals were calculated for all proportions of AIV infections (i.e., sex, age, species).

A multivariate main effects logistic regression model was also constructed to assess differences in AIV detection by using rRT-PCR by age, sex, and bird species. Species were categorized as blue-winged teal, green-winged teal, gadwall, northern shoveler, or other species. We chose the 4 species-specific categories because they represented the largest numbers of tested birds. Sample records with missing rRT-PCR results or age, sex, or species data were removed from this analysis. We analyzed all data using Intercooled Stata version 9 (Stata Corp., College Station, TX, USA).

## Results

### Sampling Overview

A total of 5,363 cloacal swab samples were collected from 33 different potential host species, including a variety of waterfowl and other wetland-associated game birds (Table A1; Table A2; and Table A3) during 3 consecutive hunting seasons (season 1: 2,171 birds; season 2: 2,424 birds; and season 3: 768 birds). Most samples (3,138 [58.5%]) were from teal (blue-winged [*Anas discors*] and green-winged [*A. crecca*]), followed by northern shovelers (*A. clypeata*; 703 [13.1%]), gadwall (*A. strepera*; 437 [8.2%]), and American wigeon (*A. americana*; 238 [4.4%]); the remaining samples (847 [15.8%]) were from a variety of other species (Table A1–3). Adults accounted for 2,759 (51.5%) samples; 1,504 (28.0%) were collected from juveniles, and 1,100 (20.5%) from birds of undetermined age. Additionally, 2,445 (45.6%) samples were from drakes, 2,262 (42.2%) from hens, and 656 (12.2%) from birds of undetermined sex.

**Table 3:** Comparison of apparent prevalence of avian influenza virus in hunter-harvested waterfowl, Texas mid–Gulf Coast, USA, September–January 2006–07, 2007–08, and 2008–09*

Hunting season	Juvenile waterfowl†				Adult waterfowl†				p value	
	No. tested	rRT-PCR	VI		No. tested	rRT-PCR	VI		rRT-PCR	VI
2006–07	518	8.30 (5.92–10.68)	3.28 (1.75–4.82)		1,081	5.46 (4.10–6.81)	0.74 (0.23–1.25)		0.029	<0.001
2007–08	763	13.80 (11.30–16.20)	5.50 (3.89–7.12)		1,189	10.51 (8.77–12.20)	3.28 (2.27–4.29)		0.030	0.022
2008–09	222	8.56 (4.88–12.24)	1.80 (0.49–4.55)		489	4.70 (2.82–6.58)	0.20 (0.01–1.13)		0.043	0.035
Total‡	1,503	11.10 (9.52–12.69)	4.06 (3.06–5.06)		2,759	1.74 (1.25–2.23)	1.74 (1.25–2.23)		<0.001	<0.001

*rRT-PCR, real-time reverse transcription–PCR; VI, virus isolation. †Values for rRT-PCR and VI are apparent prevalence, % (95% confidence interval). ‡Total = the 3 hunting seasons combined (September–January, 2006–07, 2007–08, and 2008–09).

### Subtype Prevalences

Of 4,119 samples processed for virus isolation, influenza A viruses were isolated from 134. All 9 NA subtypes (N1–9) were isolated, whereas only 9 of the 16 different HA subtypes (H1–7, 10, and 11) were isolated. Thirty-two different HA and NA subtype combinations were identified (Table A4), and for 8 isolates, either the HA (n = 7) or NA (n = 1) was not identified.

The most frequently identified HA subtypes during season 1 were H3 and H6 (8 [25.0%] and 9 [28.1%], respectively), whereas for season 2, H4 and H10 were predominant (26 [26.8%] and 17 [17.5%], respectively); the H4 subtype (4 [80.0%]) remained predominant in season 3. With respect to NA subtypes, N1 and N8 were most common in season 1 (8 [18.8%] and 10 [31.3%], respectively), whereas N6, N7, and N8 (19 [19.6%]; 16 [16.5%], and 19 [19.6%], respectively) were predominant in season 2, with N6 and N8 (2 [40.0%] each) remaining predominant in season 3. The most frequent HA and NA subtype combinations identified during season 1 were subtype H3N8 (n = 7) and H6N1 (n = 4) viruses, whereas H4N6 (n = 17), H3N8 (n = 9), and H10N7 (n = 9) viruses were the most common subtype combinations identified in season 2, and H4N6 (n = 2) and H4N8 (n = 2) were most common in season 3 (Table A4).

H7 subtype was identified by rRT-PCR during all 3 hunting seasons (n = 2, 28, and 2, respectively). Additionally, H5 subtype was detected by rRT-PCR for all 3 seasons (n = 14, 21, and 2, respectively). Yet, H5 viruses were isolated only during season 2, whereas H7 viruses were isolated during all 3 hunting seasons (Tables 1, 2). All H5 and H7 viruses were determined to be low-pathogenicity AIVs by analysis of the amino acid sequence at the HA protein cleavage site.

**Table 1:** Subtypes of avian influenza viruses isolated in the fall (September and November) from selected species during 3 consecutive hunting seasons, Texas mid–Gulf Coast, USA, 2006–07, 2007–08, and 2008–09

Species*	Subtype (no. isolated)						
	September†				November		
	2006	2007	2008		2006	2007	2008
Fulvous whistling duck (*Dendrocygna bicolor*)	–	–	–		H6N1	–	–
Mottled duck × mallard (*Anas fulvigula* × *A. platyrhynchos*)	–	–	–		–	H6N8	–
Mottled duck (*A.* *fulvigula*)	–	–	–		H6N5	–	–
Northern pintail (*A. acuta*)	–	–	–		–	H4N8	–
Northern shoveler (*A. clypeata*)	–	–	–		H2N9, H3N8, H4N2, H4N6, H4N8	H4N2, H5N2, H5N3, H6N2, H10N2, H11N9 (2)	H7N2
Teal, blue-winged (*A. discors*)	H1N1, H3N6, H3N8 (6)	H1N1 (2), H2N8, H3N4, H3N6, H3N8 (9), H4N1, H4N6 (17), H4N8 (6), H6N1, H7N1, H7N1/4, H7N7 (2), H10N7 (5)	H4N6, H4N8		H2N9, H4N2 H4N6, H4N8 H6N1 (3), H6N1/4, H6N5, H6N6, H6N8	H3N6, H5N2 (2), H5N3 (2), H7N4, H7N7 (3), H10N7, H11N9 (3)	H4N8
Teal, green-winged (*A. crecca*)	H6N2	H10N7	–		H1N1	H5N2, H7N1/4, H11N9	–

*Species selected by significance as determined by prevalence, uniqueness to the area, or native, nonmigratory species. †Teal are the only species hunted during September on the Texas mid–Gulf coast.

**Table 2:** Subtypes of avian influenza viruses isolated in the winter (December–January) from selected species during 3 consecutive hunting seasons, Texas mid–Gulf Coast, USA, 2006–07, 2007–08, and 2008–

Species*	Subtype (no. isolated)						
	December				January		
	2006	2007	2008		2007	2008	2009
Northern pintail (*Anas acuta*)	–	H10N3/7	H4N6		–	H10N3	–
Northern shoveler (*A. clypeata*)	–	H5N2, H6N2, H10N7	–		–	–	–
Teal, blue-winged (*A. discors*)	–	–	–		–	H10N3 (3)	–
Teal, green-winged (*A. crecca*)	H10N7, H11N3	–	–		H7N3	H7N3, H10N3 (2)	–

*Species selected by significance as determined by prevalence, uniqueness to the area, or native, nonmigratory species.

### Prevalence by Sex, Age, and Species

Apparent AIV prevalence did not differ significantly between hens and drakes by rRT-PCR or virus isolation during any of the 3 hunting seasons or all seasons combined (Table A5). Prevalence as determined by rRT-PCR and virus isolation differed significantly between juvenile and adult birds during the 3 hunting seasons and for all seasons combined (Table 3). However, when data were analyzed on the basis of samples for which both sex and age of the birds were known, results differed significantly between adult drakes and hens according to rRT-PCR results during season 1 and between juvenile hens and drakes by virus isolation during season 3 and for all 3 seasons combined (Table A6).

To determine whether a species effect existed for age differences, we assessed apparent AIV prevalence by age for species for which >100 samples from adult birds and >100 samples from juvenile birds were tested (i.e., blue-winged teal, green-winged teal, gadwall, and northern shoveler; Table 4; Table A7; and Table A8). When data from all 3 hunting seasons were combined, significantly more juvenile than adult birds were positive for AIV by virus isolation for 3 of the predominant host species analyzed (blue-winged teal, green-winged teal, and northern shoveler); no significant difference was observed for gadwall (Table A7–8). However, apparent AIV prevalence by rRT-PCR was significantly higher only for juvenile blue-winged teal and northern shovelers (Table A 7, 8). According to multivariate logistic regression, rRT-PCR results were associated with age and species but not with sex (Table 4).

**Table 4:** Multivariate logistic regression model to identify variables associated with a positive real-time RT-PCR result, Texas mid–Gulf Coast, USA, 2006–07, 2007–08, and 2008–09*

Variable	Odds ratio (95% CI)	p value
Sex		
Drake	1.0†	
Hen	1.07 (0.859–1.320)	0.558
Age		
Adult	1.0	
Juvenile	1.45 (1.17–1.81)	**0.001**
Species		
Other species	1.0	
Gadwall	0.407 (0.120–0.825)	**0.013**
Northern shoveler	1.51 (0.987–2.320)	0.057
Blue-winged teal	2.18 (1.52–3.13)	**<0.001**
Green-winged teal	1.12 (0.742–1.680)	0.592

*Results for a total of 4,187 samples, collected during September–January for each season. RT-PCR, reverse transcription–PCR; CI, confidence interval. **Boldface** indicates significant result. †Reference category.

Blue-winged teal and northern shovelers had the greatest diversity in subtypes, followed by green-winged teal (Tables 1, 2; Table A1–3). Nine HA (H1–7, 10, and 11) and all 9 NA (N1–9) subtypes were identified in blue-winged teal, 8 HA (H2–7, 10, and 11) and 6 NA (N2, N3, and N6–9) subtypes were identified in northern shovelers, whereas 6 HA (H1, 5–7, 10, and 11) and 6 NA (N1–4, N7, and N9) subtypes were identified in green-winged teal.

## Discussion

The Texas Gulf Coast provides winter habitat for ≈2–3 million ducks and >1 million geese (*11*). In this region, migratory waterfowl intermingle with resident wild species such as the mottled duck, and are in close contact with poultry operations and humans, primarily hunters (*15**,**17*). Recently, we reported prevalence for the first multiyear study of AIV that covered waterfowl wintering grounds along the Texas Gulf Coast (*16*), a previously understudied area. Unlike results of previous studies, we found little to no variation in apparent AIV prevalence by month within wintering seasons (September–January) with the exception of rRT-PCR during December 2007–January 2008 and virus isolation during 2005–06 and 2006–07 (*16*). Additionally, AIV prevalence, as determined by rRT-PCR or virus isolation, varied little among the 4 consecutive hunting seasons studied (September–January 2005–06 through 2008–09), except for the 2007–08 season, during which overall AIV prevalence was higher than the other 3 seasons as determined by both rRT-PCR and virus isolation (*16*). Detection of AIV at low levels throughout the wintering season supports the contention that AIV can persist in wild-bird populations through continuous circulation in a proportion of the population (*1*). The low rate of virus isolation observed in the current study (29.9% of rRT-PCR–positive samples) is consistent with findings of other studies and is not surprising (*2**,**23*). Real-time RT-PCR is considered more sensitive than virus isolation, enabling the detection of genome fragments and viruses that do not grow in embryonated chicken eggs. Also, consistent with other surveillance studies, no differences were noted in AIV prevalence based on sex, and AIV was more prevalent in juvenile birds than in adults (*1**,**7**,**23*). The latter finding supports the assumption that immunologically immature (juvenile) birds are more susceptible to AIV than are mature (adult) birds (*24**,**25*).

The most commonly identified HA and NA subtype combinations during season 1 were H3N8 and H6N1; during season 2, H3N8 remained, but it was not detected during season 3. During season 2, H4N6 and H10N7, which have been reported on the Gulf Coast (*8**,**13*), were the predominant subtype combinations; H4N6 also was detected during season 3. The annual variations in AIV subtype prevalence observed in this study show the need for continued annual surveillance in domestic and migratory avian species, particularly in areas of high poultry and waterfowl density, such as the Texas Gulf Coast (*15*).

Outbreaks of H5 AIV have been documented previously in Texas. In 1993, an outbreak of H5N2 occurred in emus, in 2002 H5N3 was detected in chickens, and in 2004 highly pathogenic avian influenza virus (H5N2) was reported in a commercial poultry operation (*26**–**28*). We isolated subtype H5N2 and H5N3 viruses from apparently healthy free-roaming waterfowl only during season 2. Although no data are available on subtypes circulating in waterfowl on the Texas coast before the 3 outbreaks noted above, our data document the presence of these subtypes in migratory waterfowl near commercial poultry operations (*15*). Molecular characterization of the subtype H5N2 and H5N3 viruses we isolated should help clarify the relation between these viruses and those isolated from commercial species.

Our isolation of AIVs from resident (nonmigratory) mottled ducks and mottled duck/mallard hybrids suggests AIV transmission on the wintering ground and is consistent with previous reports (*13*). Mallards interbreed with mottled ducks and are sister species phylogenetically (*29*). Before the isolation of H6 AIVs from a mottled duck/mallard hybrid in November 2006 and a mottled duck in November 2007, we isolated H6 subtypes from migratory teals and northern shovelers (September and November 2006 and 2007). Additional support for AIV transmission on wintering grounds included isolation of an H6 virus from a fulvous whistling duck, a species that breeds on the Texas–Louisiana coast and leaves during late summer to winter further south in Mexico; nearly all whistling ducks are gone by late January (*17*). Although circulation of AIVs within fulvous whistling ducks, mottled ducks, and mottled duck hybrids throughout the year cannot be ruled out, such circulation seems unlikely. Hanson et al. were unable to isolate AIVs from mottled ducks collected on the Texas Gulf Coast during August (*9*); additionally, we did not detect AIV by rRT-PCR in samples collected during June–August 2007 (n = 155, S. Rollo et al., unpub. data), which suggests that these viruses are not readily circulating in these resident populations during summer. Genetic characterization of these H6 isolates will help determine whether these isolates are related and help clarify the role of waterfowl wintering grounds in the transmission and perpetuation of AIVs in nature. Further studies focused on AIV prevalence and immune responses to AIV in these resident populations also are needed to clarify the maintenance and transmission of AIVs in the wintering grounds.

Before singling out a particular species on which to focus surveillance efforts, one must consider the technique used for subject selection (hunter-harvest vs. live-capture) as well as the area under study (e.g., breeding grounds vs. wintering grounds; fresh water vs. salt water) and which populations are prevalent within the study areas. Mallards have become a primary species of interest not only because of their susceptibility to H5 and H7 subtypes but also because of their abundance and relative ease of capture (*2**,**17**,**23**,**30**–**32*). During our study, few mallard samples were collected because most Texas mallards winter in the playa lakes and sorghum fields of the Texas Panhandle with few (<4%) wintering along the Gulf Coast (*17*). Our data indicate that mallards, although appropriate focal species for AIV monitoring in some portions of North America, are not as suitable as blue-winged teal or northern shoveler in other regions, such as the Texas mid–Coast (*8**,**9**,**13*). In many studies that found mallards as a high-prevalence species for AIV infection, they were captured live for testing and dominated the samples (*2**,**23*). The few studies in which other species were more frequently sampled and tested positive for AIV were conducted on hunter-harvested waterfowl (*8**,**13**,**33*).

Our study supports the consensus that dabbling ducks are more likely than diving ducks to be positive for AIV; however, as others have documented, not all dabbling ducks are equally likely to be AIV positive (*2**,**23**,**33*). We found blue-winged teal to be the species with the highest prevalence, followed by northern shoveler and green-winged teal. Gadwalls, also a dabbling duck from which we collected substantial numbers of samples, were the least likely to test positive for AIV. Blue-winged teal are generally the first ducks to fly south in the fall, first arriving on wintering grounds in September, and the last to pass through Texas in late February–March on their return north (*17*). They also make exceedingly long flights compared with other dabbling ducks between feeding and resting areas during migrations (*17*). On the other hand, gadwalls are short-distance migrants and migrate later, generally beginning their southward migration in early September and their return north starting in February (*17*). The physiologic demands of long-distance migration can suppress the immune system (*34*); thus, blue-winged teal might be more susceptible to infection than some other dabbling ducks because of their long-distance migration. More extensive studies are needed incorporating more ecologic factors such as food resources, body mass, and immune status to more fully understand how AIV persists in nature and why the prevalence of AIV is higher in particular species.

Although our samples were not collected probabilistically (i.e., the samples reflect hunters’ choices, as well as the relative abundance of each species), use of hunter-harvested waterfowl was convenient for obtaining large number of samples with which to estimate the prevalence of AIV subtypes carried by waterfowl in the Gulf Coast of Texas. In addition, because hunters have been identified as the human population most at risk for exposure to AIV (*35*) and antibodies to H11 subtype have been identified in hunters and wildlife professionals (*36*), continued monitoring of AIV in waterfowl and humans exposed to them should provide useful information about the prevalence and significance of wild animal-to-human transmission.

AIV surveillance studies over time in the same region are critical, particularly in understudied areas. Although studies in areas of low AIV prevalence are inconvenient because of the large sample sizes required to isolate substantial numbers of AIVs, such surveys are critical to gain more knowledge of the ecology of influenza viruses. Our data contribute temporal information about AIV prevalence and subtype diversity for a historically understudied area of North America, the waterfowl wintering grounds of the Texas Gulf Coast.

## Appendix

**Table A1:** Apparent prevalence of avian influenza virus in various waterfowl and wetland-associated game bird species as determined by VI and real-time RT-PCR from cloacal swabs collected from hunter-harvested waterfowl, Texas mid–Gulf Coast, USA, September 2006–January 2007*

Species	No. tested	Real-time RT-PCR,† no. (%)	VI,† no. (%)	Isolate‡
American wigeon (*Anas americana*)	171	4 (2.3)	0	–
Fulvous whistling duck (*Dendrocygna bicolor*)	18	2 (11.1)	1 (5.6)	H6N1
Lesser scaup (*Aythya affinis*)	60	1 (1.7)	1 (1.7)	H10N7
Mallard (*Anas platyrhynchos*)	5	1 (20.0)	0	–
Mottled duck (*Anas fulvigula*)	33	2 (6.1)	1 (3.0)	H6N5
Northern pintail (*Anas acuta*)	72	5 (6.9)	0	–
Northern shoveler (*Anas clypeata*)	360	23 (6.4)	5 (1.3)	H2N9, H3N8, H4N2, H4N6, H4N8
Redhead (*Aythya amercana*)	51	2 (3.9)	0	–
Ring-necked duck (*Aythya collaris*)	35	1 (2.9)	0	–
Ruddy duck (*Oxyura jamaicensis*)	31	2 (6.5)	0	–
Teal, blue-winged (*Anas discors*)	610	65 (10.7)	19 (3.1)	H1N1, H2N9, H3N6, H3N8 (6), H4N2, H4N6, H4N8, H6N1 (3), H6N1/4, H6N5, H6N6, H6N8
Teal, green-winged (*Anas crecca*)	358	31 (8.7)	5 (1.4)	H1N1, H6N2, H7N3, H10N7, H11N3
Snow goose (*Chen eaerulescens*)	46	2 (4.4)	0	–
Total§	2,171	141 (6.5)	32 (1.5)	–

*VI, virus isolation; RT-PCR, reverse transcription–PCR. †Number positive (apparent prevalence). Numbers and apparent prevalences for VI are after RT-PCR result. ‡Isolates typed by the National Veterinary Services Laboratory. Included are VI that were RT-PCR negative on the original sample. §Other species sampled that were negative for AI by RT-PCR and VI, number sampled: American coot (*Fulica americana*), 25; black-bellied whistling duck (*Dendrocygna autumnalis*), 6; bufflehead (*Bucephala albeola*), 2; canvasback (*Aythya valisineria*), 16; cinnamon teal (*Anas cyanoptera*), 1; common goldeneye (*Bucephala clangula*), 1;common moorhen (*Gallinula chloropus*), 1; common snipe (*Gallinago gallinago*), 1; gadwall (*Anas strepera*), 247; greater white-front goose (*Anser albifrons*), 3; hooded merganser (*Laphodytes cucullatus*), 11; mottled duck x mallard hybrid (*Anas fulvigula x Anas platyrhynchos*), 1; Ross’s goose (*Anser albifrons*), 2; sandhill crane (*Grus candensis*), 1; wood duck (*Aix sponsa*), 1; and unidentified teal, 2.

**Table A2:** Apparent prevalence of avian influenza virus in various waterfowl and wetland-associated game bird species as determined by VI and real-time RT-PCR from cloacal swabs collected from hunter-harvested waterfowl, Texas mid–Gulf Coast, USA, September 2007–January 2008*

Species	No. tested	Real-time RT-PCR,† no. (%)	VI,† no. (%)	Isolate‡
American wigeon (*Anas americana*)	51	8 (15.7)	0	–
Fulvous whistling duck (*Dendrocygna bicolor*)	14	1 (7.1)	0	–
Gadwall (*Anas strepera*)	160	10 (6.3)	1 (0.6)	H6N1
Mottled duck (*Anas fulvigula*)	26	1 (3.9)	0	–
Mottled duck x Mallard (*A. fulvigula x A. platyrhynchos*)	2	1§	1§	H6N8
Northern pintail (*Anas acuta*)	62	13 (21.0)	3 (4.8)	H4N8, H10N3, H10N3/7
Northern shoveler (*Anas clypeata*)	239	38 (15.9)	10 (4.2)	H4N2, H5N2 (2), H5N3, H6N2 (2), H10N2, H10N7, H11N9 (2)
Ring-necked duck (*Aythya collaris*)	17	1 (5.9)	0	–
Ruddy duck (*Oxyura jamaicensis*)	36	2 (5.6)	1 (2.8)	H2N3
Teal, blue-winged (*Anas discors*)	1,213	155 (12.8)	73 (6.0)	H1N1 (2), H2N8, H3N4, H3N6 (2), H3N8 (9), H4N1, H4N6 (17), H4N8 (6), H5N2 (2), H5N3 (2), H6N1, H7N1, H7N1/4, H7N4, H7N7 (5), H10N?, H10N3 (2), H10N3/7, H10N7 (7), H11N9 (3)
Teal, cinnamon (*Anas cyanoptera*)	2	1§	1§	H7N3
Teal, green-winged (*Anas crecca*)	464	38 (8.2)	7 (1.5)	H5N2, H7N1/4, H7N3, H10N3, H10N3/7, H10N7, H11N9
Snow goose (*Chen eaerulescens*)	43	3 (7.0)	0	–
Total¶	2,424	272 (11.2)	97 (4.0)	–

*VI, virus isolation; RT-PCR, reverse transcription–PCR. †Number positive (apparent prevalence). Numbers and apparent prevalence for VI are after RT-PCR result. ‡Isolates typed by the National Veterinary Services Laboratory. Seven isolates were confirmed as avian influenza but were unable to be subtyped. Included are virus isolates that were RT-PCR negative on the original sample. §Apparent prevalence not calculated due to the small sample size. ¶Other species sampled that were negative for AI by RT-PCR and VI, number sampled: American coot (*Fulica americana*), 27; Black-bellied whistling duck (*Dendrocygna autumnalis*), 9; Canvasback (*Aythya valisineria*), 3; Common snipe (*Gallinago gallinago*), 2; Greater white-front goose (*Anser albifrons*), 3; Hooded merganser (*Laphodytes cucullatus*), 3; Least grebe (*Tachybaptus dominicus*), 1; Lesser Scaup (*Aythya affinis*), 26; Mallard (*Anas platyrhynchos*), 4; Redhead (*Aythya amercana*), 3; Ross’s goose (*Anser albifrons*), 5; Sandhill crane (*Grus candensis*), 8; and unidentified teal, 1.

**Table A3:** Apparent prevalence of avian influenza virus in various waterfowl and wetland-associated game bird species as determined by VI and real-time RT-PCR from cloacal swabs collected from hunter-harvested waterfowl, Texas mid–Gulf Coast, USA, September 2008–January 2009*

Species	No. tested	Real-time RT-PCR,† no. (%)	VI,† no. (%)	Isolate‡
American wigeon (*Anas americana*)	16	1 (6.3)	0	–
Northern pintail (*A. acuta*)	28	2 (7.1)	1 (3.6)	H4N6
Northern shoveler (*A. clypeata*)	104	5 (4.8)	1 (1.0)	H7N2
Teal, blue-winged (*A. discors*)	176	21 (11.9)	3 (1.7)	H4N6, H4N8 (2)
Teal, green-winged (*A. crecca*)	314	13 (4.1)	0	–
Total§	768	42 (5.5)	5 (0.7)	–

*VI, virus isolation; RT-PCR, reverse transcription–PCR. †Number positive (apparent prevalence). ‡Isolates typed by the National Veterinary Services Laboratory. §Other species sampled that were negative for AI by RT-PCR and VI, number sampled: Common ground dove (*Columbina passerina*), 1; Gadwall (*Anas strepera*), 30; Greater white-front goose (*Anser albifrons*), 6; Hooded merganser (*Laphodytes cucullatus*), 4; Lesser Scaup (*Aythya affinis*), 1; Mallard (*Anas platyrhynchos*), 4; Mottled duck (*Anas fulvigla*), 23; Mottled duck x Mallard hybrid (*Anas fulvigula x Anas platyrhynchos*), 1; Ring-necked duck (*Aythya collaris*), 1; Ross’s goose (*Anser albifrons*), 1; Sandhill crane (*Grus candensis*), 1; Snow goose (*Chen eaerulescens*), 56; and Wood duck (*Aix sponsa*), 1.

**Table A4:** Hemagglutinin and neuramindase subtype combinations for all avian influenza viruses isolated from hunter-harvested waterfowl, Texas mid–Gulf Coast, USA, September–January 2006–07, 2007–08, and 2008–09

Neuraminidase	Hunting season	Hemagglutinin*									
		1	2	3	4	5	6	7	10	11	Total
1	2006–07	2	–	–	–	–	5	–	–	–	7
	2007–08	2	–	–	1	–	2	3	–	–	8
2	2006–07	–	–	–	2	–	1	–	–	–	3
	2007–08	–	–	–	1	5	2	–	1	–	9
	2008–09	–	–	–	–	–	–	1	–	–	1
3	2006–07	–	–	–	–	–	–	1	–	1	2
	2007–08	–	1	–	–	3	–	2	7	–	13
4	2006–07	–	–	–	–	–	–	–	–	–	0
	2007–08	–	–	1	–	–	–	1	–	–	2
5	2006–07	–	–	–	–	–	2	–	–	–	2
	2007–08	–	–	–	–	–	–	–	–	–	0
6	2006–07	–	–	1	2	–	1	–	–	–	4
	2007–08	–	–	2	17	–	–	–	–	–	19
	2008–09	–	–	–	2	–	–	–	–	–	2
7	2006–07	–	–	–	–	–	–	–	2	–	2
	2007–08	–	–	–	–	–	–	5	9	–	14
8	2006–07	–	–	7	2	–	1	–	–	–	10
	2007–08	–	1	9	7	–	1	–	–	–	18
	2008–09	–	–	–	2	–	–	–	–	–	2
9	2006–07	–	2	–	–	–	–	–	–	–	2
	2007–08	–	–	–	–	–	–	–	–	6	6
Total	2006–07	2	2	8	6	0	10	1	2	1	32
	2007–08	2	2	12	26	8	5	11	17	6	89†
	2008–09	0	0	0	4	0	0	1	0	0	5
	Total	4	4	20	36	8	15	13	19	7	126

*No H8, H9, or H12–16 were identified in this study. †Eight isolates not recorded because of inability to subtype the hemagglutinin and/or neuraminidase. All isolates included regardless of real-time reverse transcription–PCR result.

**Table A5:** Comparison of apparent prevalence of avian influenza virus by sex and detection method in hunter-harvested waterfowl, Texas mid–Gulf Coast, USA, September–January 2006–07, 2007–08, and 2008–09

Hunting season	Hen				Drake			p value for rRT-PCR, VI
	No. tested	Real-time RT-PCR*	VI†		No. tested	Real-time RT-PCR*	VI	
2006–07	967	7.14 (5.51–8.76)	1.24 (0.54–1.94)		1,083	6.10 (4.67–7.53)	1.48 (0.76–2.20)	0.343, 0.645
2007–08	895	13.30 (11.10–15.50)	4.69 (3.31–6.08)		1,059	10.70 (8.89–12.60)	3.77 (2.62–4.92)	0.084, 0.312
2008–09	400	5.00 (2.86–7.14)	0.25 (0.01–1.38)		303	7.26 (4.34–10.18)	1.32 (0.36–3.35)	0.210, 0.171
Total‡	2,262	9.20 (8.00–10.39)	2.39 (1.76–3.02)		2,445	8.26 (7.17–9.35)	2.41 (1.80–3.02)	0.255, 0.956

*RT-PCR, reverse transcription–PCR. Apparent prevalence, % (95% confidence interval). †VI, virus isolation. Apparent prevalence, % (95% confidence interval). ‡Total = all 3 seasons combined (September−January 2006–07, 2007–08, and 2008−09).

**Table A6:** Apparent prevalence of avian influenza virus by age, sex, and detection method in hunter-harvested waterfowl, Texas mid–Gulf Coast, USA, September–January 2006–07, 2007–08, and 2008–09

Age/sex	Hunting season											Total, 2006–09		
	2006–07				2007–08				2008–09					
	No. tested	Real-time RT-PCR*	VI†		No. tested	Real-time RT-PCR*	VI		No. tested	Real-time RT-PCR*	VI	No. tested	Real-time RT-PCR*	VI
Adult														
Hen	483	34 (7.0)	5 (1.0)		468	55 (11.8)	20 (4.3)		259	10 (3.9)	1 (0.4)	1,210	99 (8.2)	26 (2.2)
Drake	580	24 (4.1)	3 (0.5)		704	69 (9.8)	19 (2.7)		221	13 (5.9)	0	1,505	106 (7.0)	22 (1.5)
Total	1,063	58 (5.5)	8 (0.8)		1,172	124 (10.6)	39 (3.3)		480	23 (4.8)	1 (0.2)	2,715	205 (7.6)	48 (1.8)
p value	–	**0.038**	0.33		–	0.288	0.141		–	0.301	0.355	–	0.264	0.177
Juvenile														
Hen	250	15 (6.0)	5 (2.0)		409	60 (14.7)	20 (4.9)		136	10 (7.4)	0	795	85 (10.7)	25 (3.1)
Drake	254	27 (10.7)	12 (4.7)		344	45 (13.1)	20 (5.8)		80	9 (11.3)	4 (5.0)	678	81 (12.0)	36 (5.3)
Total	504	42 (8.3)	17 (3.4)		753	105 (13.9)	40 (5.3)		216	19 (8.8)	4 (1.9)	1,473	166 (11.3)	61 (4.1)
p value	–	0.060	0.090		–	0.531	0.573		–	0.329	**0.008**	–	0.448	**0.038**

*RT-PCR, reverse transcription–PCR. No. positive (apparent prevalence, %). †VI, virus isolation. No. positive (apparent prevalence, %). **Boldface** indicates significance.

**Table A7:** Comparison of apparent prevalence of avian influenza virus in blue-winged and green-winged teal from hunter-harvested waterfowl, Texas mid–Gulf Coast, USA, September–January 2006–07, 2007–08, and 2008–09

Age/sex	Blue-winged teal				Green-winged teal		
	No. tested	Real-time RT-PCR*	VI†		No. tested	Real-time RT-PCR*	VI†
Adult	910	11.00 (8.97–13.04)	3.85 (2.60–5.10)		790	6.33 (4.63–8.03)	0.38 (0.08–1.11)
Juvenile	620	15.48 (12.64–18.33)	6.94 (4.94–8.94)		251	7.57 (4.30–10.84)	1.99 (0.26–3.72)
p value		**0.010**	**0.007**			0.491	**0.011**
Hen	772	13.73 (11.30–16.16)	4.40 (2.96–5.85)		548	6.57 (4.50–8.64)	0.91 (0.12–1.71)
Drake	894	11.30 (9.22–13.37)	4.92 (3.50–6.34)		581	7.75 (5.57–9.92)	0.86 (0.11–1.61)
p value		0.133	0.618			0.444	0.926

*RT-PCR, reverse transcription–PCR. Apparent prevalence, % (95% confidence interval). †VI, virus isolation. Apparent prevalence, % (95% confidence interval). **Boldface** indicates significance.

**Table A8:** Apparent prevalence of avian influenza virus in gadwall and northern shoveler from hunter-harvested waterfowl, Texas mid–Gulf Coast, USA, September–January 2006–07, 2007–08, and 2008–09

Age/sex	Gadwall				Northern shoveler		
	No. tested	Real-time RT-PCR*	VI†		No. tested	Real-time RT-PCR*	VI†
Adult	233	2.15 (0.29–4.01)	0.43 (0.01–2.37)		363	7.16 (4.51–9.82)	1.38 (0.18–2.58)
Juvenile	144	3.47 (0.48–6.46)	0		258	12.40 (8.38–16.43)	3.88 (1.52–6.23)
p value		0.436	0.431			**0.027**	**0.046**
Hen	221	1.36 (0.28–3.92)	0.45 (0.01–2.50)		373	10.99 (7.82–14.17)	2.68 (1.04–4.32)
Drake	216	3.24 (0.88–5.60)	0		330	7.58 (4.72–10.43)	1.52 (0.20–2.83)

*RT-PCR, reverse transcription–PCR. Apparent prevalence, % (95% confidence interval). †VI, virus isolation. Apparent prevalence, % (95% confidence interval). **Boldface** indicates significance.
